# The Swedish welfare state under threat: new rules and new inequalities

**DOI:** 10.1016/j.lanepe.2026.101692

**Published:** 2026-04-29

**Authors:** Amy Heshmati, Helena Honkaniemi, Sol P. Juárez

**Affiliations:** a Centre for Health Equity Studies (CHESS), Stockholm University/Karolinska Institutet, Stockholm, Sweden; b Department of Public Health Sciences, Stockholm University, Stockholm, Sweden; c Department of Global Public Health, Karolinska Institutet, Stockholm, Sweden; d Stockholm University Demography Unit, Stockholm University, Sweden

Strong social protections are essential for reducing health inequalities in society. Sweden has long been recognised for having one of the world’s most generous social welfare systems, which to date has been available to all residents regardless of their length of residency or citizenship status, but even this system has not been immune to political change.^1^

The Swedish government has proposed a reform that would introduce new residence-based rules for accessing social security benefits. Although framed as a way to incentivise employment and reduce long-term benefit dependency, the reform is expected to disproportionately affect migrant residents by excluding them from core welfare protections. As a result, these rules are likely to exacerbate the well-documented health and social inequalities between people with and without migrant backgrounds in Sweden.^2^ By conditioning access to social protection on duration of residence or prior income, the reform introduces mechanisms of selective inclusion and exclusion that disproportionately affect individuals in more precarious socioeconomic positions.

If the government’s proposal is passed, migrant residents would need to live in Sweden for at least five years within a fifteen-year period in order to qualify for social benefits.^3^ This requirement would apply to a range of essential supports, including child allowance, housing benefits, sickness and parental leave benefits at the basic and lowest compensation levels. However, the proposal also includes a faster track to qualification based on earnings, requiring either a monthly income of SEK 40 032 (GBP 3260) for six consecutive months (above the median monthly salary in Sweden) or SEK 20 850 (GBP 1700) for 12 of the previous 24 months.^3^ This track effectively denies social protections to those migrants who need them the most. Such criteria risk generating cumulative disadvantage, whereby exclusion from social protection contributes to deteriorating health and reduced labour market participation, further reinforcing exclusion. These developments occur alongside ongoing changes in the Swedish healthcare system, including an expansion of privately delivered but publicly financed care, as well as increasing reliance on private health insurance. Taken together, these changes represent a shift away from the long-standing principle of care and social protection on equal terms according to need, as established in Swedish health policy. Consequently, this reform would mark a significant shift away from Sweden’s long-standing universal welfare model, aligning with the country’s recent anti-migration policies that restrict entry and limit residents’ rights. A decision on the proposal is expected on May 20, 2026, and if approved, the reform will be implemented on January 1, 2027.^4^

As foreign researchers working in the field of health inequalities, we urge a sensitive political response that does not disregard the insurmountable empirical evidence that shows that restrictive policies limiting migrants’ opportunities not only make integration harder, but also increase inequalities and weaken social cohesion, with negative public health consequences for society as a whole.^5^ If implemented, these reforms risk not only increasing health inequalities but also weakening key institutional features that have historically supported equity in the Swedish welfare model.

## Contributors

AH & SPJ conceived the idea and drafted the first version of the manuscript, with substantial contributions from HH. All authors reviewed and approved the final version.

## Declaration of interests

We declare no competing interests.
